# Gustatory Function of Patients With and Without Cholesteatoma Undergoing Middle Ear Surgery

**DOI:** 10.1177/00034894221129911

**Published:** 2022-10-26

**Authors:** Aline Sophie Neumann, Michael B. Soyka, Elisabeth J. Rushing, Christof Röösli

**Affiliations:** 1 Department of Otorhinolaryngology, Head and Neck Surgery, University Hospital Zurich, University of Zurich, Zürich, Switzerland; 2 Department of Neuropathology, University Hospital Zurich, University of Zurich, Zürich, Switzerland

**Keywords:** cholesteatoma, chorda tympani nerve, gustatory function, middle ear surgery, otology

## Abstract

**Objective:**

To compare measured and perceived taste function before and after surgery of patients with chronic otitis media with cholesteatoma (OMCC) to patients without cholesteatoma (patients with chronic suppurative otitis media [CSOM] and patients with lateral skull base lesions [LSB]).

**Methods:**

This prospective cohort study included 29 patients undergoing surgery for unilateral OMCC. The chorda tympani nerve (CTN) was resected in 8 of these patients. Fourteen patients undergoing surgery for unilateral CSOM and 5 patients undergoing surgery for unilateral LSB (with CTN resection) served as the comparison group. Taste function was measured using taste strips on both sides of the tongue before surgery, 2 weeks postoperatively and 3 months postoperatively. The affected side of the tongue was compared to the unaffected side. A questionnaire on taste perception was completed at each visit.

**Results:**

Preoperatively, cholesteatoma patients showed higher taste strip scores than non-cholesteatoma patients, indicating a larger difference between the healthy and affected sides of the tongue. Despite this difference in measured taste function few cholesteatoma patients reported taste alteration before surgery (3/29 [10.3%]). Postoperatively, patients with CTN resection (OMCC patients with CTN resection and LSB patients) showed a decreased measured taste function. Subjectively, only approximately 20% of these patients reported taste alteration 3 months postoperatively.

**Conclusions:**

Before surgery, cholesteatoma patients displayed an impaired measured taste function compared to patients without cholesteatoma (CSOM, LSB). Subjectively this was often unnoticed. After surgery, despite removal of the CTN and consequent reduction of measured taste function, few patients reported taste alteration and subjective taste perception was seen to be improving. In regards to middle ear surgery, perceived taste function does not seem to reflect measured gustatory function.

## Introduction

The chorda tympani nerve (CTN) carries afferent fibers for gustatory innervation of the anterior two-thirds of the tongue. After branching off from the facial nerve, the CTN runs through the posterior colliculus into the tympanic cavity.^1^ The nerve traverses the cavity across the tympanic membrane and runs between the incus and the malleus to exit through the petrotympanic fissure.^1^ As the CTN travels uncovered through the middle ear cavity, it is susceptible to trauma such as stretching, drying, injury, or severing during middle ear surgery. Altered taste function after surgery has been reported for patients suffering from chronic otitis media with cholesteatoma (OMCC) with normalization of taste function 1 year postoperatively.^2^ However, postoperative taste disturbances seem to be perceived inconsistently. Saito et al^3^ reported that approximately 3% of patients complained of postoperative taste disorders even though the CTN was scarcely touched during surgery. In contrast, after severing the CTN, only 40% to 60% of patients reported postoperative taste disturbances.^3,4^

The underlying pathological processes in the middle ear, most commonly OMCC, seem to influence nerve function as well. The inflammatory process in OMCC is thought to affect the nerve by exposing it to compression and inflammatory reactions. Several studies have demonstrated that altered taste function was present preoperatively.^5,6^ Histopathological studies of the CTN have indicated various changes of the nerve that might explain these taste alterations.^7,8^

The primary objective of the present study was to compare measured taste function before and after surgery of patients with cholesteatoma (OMCC) to that of patients without cholesteatoma (patients with chronic suppurative otitis media [CSOM] and patients with lateral skull base lesions [LSB]). Secondary objectives were to analyze the subjective taste perception, the extension of the cholesteatoma, and the histology of the severed CTNs.

The study protocol was approved by the local ethics committee. It was conducted in compliance with formalities of the independent ethical commission and the current Helsinki Declaration. Informed consent was obtained from all participants before enrollment.

## Materials and Methods

Patients undergoing surgery for OMCC served as the study group. In some patients, the CTN had to be resected. Patients undergoing surgery for CSOM with CTN preservation or for LSB (chondroma of the jugular foramen, intravestibular or intracochlear schwannoma that did not extend to the middle ear or affect facial nerve function) with removal of the CTN served as the comparison group. Inclusion criteria were patient age between 18 and 75 years and presence of unilateral disease. Exclusion criteria were pregnancy, radiation therapy of the head and neck, and presence of disease that might affect taste perception (chronic renal failure, cirrhosis of the liver, xerostomia, and depression). Patients were included from November 2019 through March 2021.

All patients underwent gustatory testing separately on each side of the tongue and bilateral olfactory testing. In addition, patients completed the validated chemical senses questionnaire (CSQ) on taste and smell disturbance before surgery, 2 weeks postoperatively, and 3 months postoperatively.^9^ The cholesteatoma was classified intraoperatively using the “ChOLE” classification.^10^ Removed CTNs were analyzed for histopathological changes by 1 neuropathologist blinded to the results of gustatory testing.

### Gustatory Function

*Measured taste function (taste strip score)*: Gustatory function was tested using taste strips infused with 4 taste qualities as described by Mueller et al.^11^ One hour before testing, participants were asked not to eat, drink, smoke, or chew gum. Nineteen taste strips (16 strips with 4 concentrations of each taste quality and 3 blank strips) were presented in randomized order separately on each side of the anterior two-thirds of the tongue. Patients were asked to keep their mouths open and to identify the taste using 1 of 5 descriptors (sour, sweet, salty, bitter, or tasteless). Between the strips, patients rinsed their mouths with water. Each correctly identified taste strip (not including tasteless) was counted as 1 point for a maximum score of 16 points. To reduce the effect of interindividual variance in patients with unilateral disease, the difference in taste scores between the healthy and affected sides of the tongue was calculated for each patient. This was labeled taste strip score.

*Subjective taste perception*: The CSQ includes 6 questions about taste sensation.^9^ For this study, 3 questions were analyzed: (1) Patients were asked about problems with their ability to taste sweet, sour, salty, or bitter foods in the last 12 months. This was labeled 1-year taste alteration. (2) A decreased ability to taste now compared to taste at 25 years of age was labeled long-term taste alteration. These 2 questions were combined as 1-year and long-term taste alteration. (3) Experiencing a persistent taste or other sensation in the past 12 months was labeled phanto-/parageusia.

### Olfactory Function

Olfactory function was tested to rule out taste disturbances due to nasal pathology. This test consisted of 12 pens filled with various fragrances.^12^ After application of a decongestant nasal spray, 1 pen was presented and patients were asked to identify the fragrance from 4 descriptors. Each correctly identified odor was counted as 1 point for a maximum score of 12 points. Nine or more points was considered a normal smell function.

### “ChOLE” Classification

The ear surgeon used the “ChOLE”-classification to stage the cholesteatoma intraoperatively.^10^ Cholesteatomas were rated by extension (1-4 points), by status of the ossicular chain at the end of surgery (0-4 points), by life-threatening complications (0, 2, or 4 points), and by degree of mastoid pneumatization and Eustachian tube ventilation (0-2 points). The sum was classified as stages I to III. Disease extension was analyzed to assess the effect of the cholesteatoma on the CTN.

### Histological Examination

Resected CTNs were examined using light microscopy. Specimens were received in 3.9% glutaraldehyde in 0.1 M sodium phosphate buffer, pH 7.4. After orienting the nerves in the transverse plane, tissues were embedded in Epon and semi-thin sections (500 nm) were cut and stained with toluidine blue. Previously described histopathological changes of the CTN served as the basis of the work-up.^7,8^ Assessed were signs of axonal atrophy, degeneration, fibrosis, and demyelination.

### Statistical Analysis

Statistical analysis was performed using SPSS software version 26.0 (IBM, Armonk, NY, USA). Descriptive statistics are presented as medians (mdn), means, and standard deviations (SD). The taste strip scores did not show a normal distribution. Therefore, the Gaussian distribution could not be assumed and the mdn was used to report data because it is more robust and sensible for skewed distribution. For comparison, the Mann-Whitney *U* test was used. The Friedman test was used to assess measured taste function over time. A Bonferroni correction was applied when appropriate. Spearman’s rank correlation was used to examine a correlation between taste strip scores and extension of the cholesteatoma. A 2-sided *P*-value of .05 was considered significant. Completed visits for participants lost to follow-up were included in the analysis. However, only patients who completed all visits were included in the analysis of measured taste function over time.

## Results

**Figure 1.**
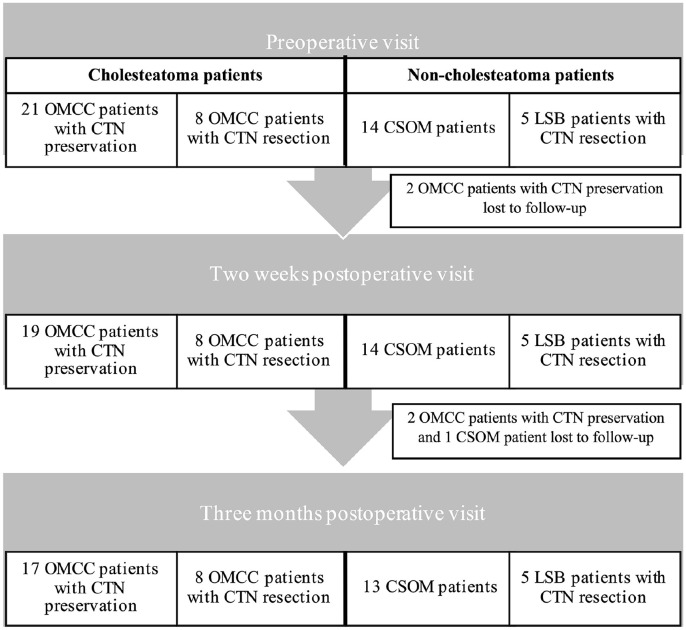
The number of patients per group and per visit. Abbreviations: CSOM, chronic suppurative otitis media; CTN, chorda tympani nerve; LSB, lateral skull base lesions; OMCC, chronic otitis media with cholesteatoma.

The final study population consisted of 48 patients. Twenty-nine patients underwent surgery for OMCC, and the CTN was resected in 8 patients. Fourteen patients underwent surgery for CSOM and 5 patients underwent surgery for LSB with CTN resection. Four OMCC patients with CTN preservation and 1 CSOM patient withdrew from the study. The number of patients per group and per visit is presented in Figure 1.

Patient characteristics are shown in Table 1. Patients were seen a mean of 1.6 day (SD 1.2) before surgery, 2 weeks (mean 14.0 days, SD 6.4) after surgery, and 3 months (mean 100.4 days, SD 25.2) after surgery.

**Table 1.** Patient Characteristics.

	Time point	Cholesteatoma patients		Non-cholesteatoma patients		*P*-value
		OMCC with CTN preservation, n = 21	OMCC with CTN resection, n = 8	CSOM, n = 14	LSB, n = 5	
Gender (n [%])						
Men	Pre op	10 [47.6%]	8 [100%]	7 [50%]	3 [60%]	.050
Age (mean [SD])	Pre op	40.2 [12.6]	39.3 [12.7]	40.6 [12.6]	48.6 [11.1]	0.579
Smoker (n [%])	Pre op	7 [33.3%]	4 [50%]	7 [50%]	0 [0%]	.197
Affected ear (n [%])						
Right	Pre op	11 [52.4%]	3 [37.5%]	10 [71.4%]	2 [40%]	.406
Smell score (mean [SD])	Pre op	10.3 [1.0]	11.0 [0.8]	10.3 [2.6]	10.2 [1.1]	.257

Participants’ age and smell scores are presented as mean and standard deviation. Participants’ gender, smoking status, and side of the affected ear are shown as numbers and percentages.

Abbreviations: CSOM, chronic suppurative otitis media; CTN, chorda tympani nerve; LSB, lateral skull base lesions; n, number; pre op, preoperatively; SD, standard deviation; OMCC, chronic otitis media with cholesteatoma.

### Preoperative Gustatory Function

**Figure 2.**
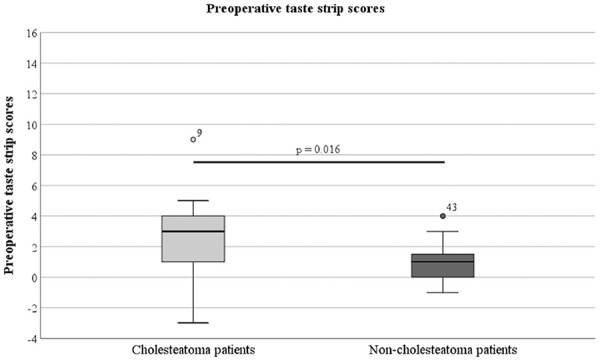
The preoperative taste strip scores of the cholesteatoma patients and the non-cholesteatoma patients.

*Measured taste function (taste strip score)*: Before surgery, taste strip scores were compared between cholesteatoma patients (OMCC) and non-cholesteatoma patients (CSOM, LSB). Cholesteatoma patients showed higher taste strip scores (mdn = 3.0) than non-cholesteatoma patients (mdn = 1.0, *P* = .016), indicating a larger difference between the healthy and affected sides of the tongue. The preoperative taste strip scores are shown in Figure 2.

*Subjective taste perception*: The preoperative results of the questionnaire are shown in Table 2a. Contrary to the taste strip scores few cholesteatoma patients reported taste alteration preoperatively (3/29 [10.3%]). Approximately 20% of non-cholesteatoma patients experienced taste alteration.

**Table 2a.** Preoperative Results of the Taste Questionnaire.

	Time point	1-Year and long-term taste alteration (n [%])	Phanto-/parageusia (n [%])	Combination of these taste changes (n [%])
Cholesteatoma patients, n = 29	Pre op	3 [10.3%]	—	1 [3.4%]
Non-cholesteatoma patients, n = 19	Pre op	4 [21.1%]	2 [10.5%]	1 [5.3%]

The results of the taste questionnaire before surgery are shown as numbers and percentages.

Abbreviations: n, number; pre op, preoperatively.

### Postoperative Gustatory Function

*Measured taste function (taste strip score)*: Postoperatively, the taste strip scores of OMCC patients with CTN preservation were compared to those of CSOM patients. Two weeks after surgery, OMCC patients with CTN preservation (mdn = 5.0) demonstrated higher taste strip scores than CSOM patients (mdn = −0.5). This difference was seen to be statistically significant (*P* = .003). Three months postoperatively, the taste strip scores of OMCC patients with CTN preservation (mdn = 2.0) remained to be higher than those of CSOM patients (mdn = 1.0). However, a difference was no longer detectable (*P* = .432).

**Figure 3.**
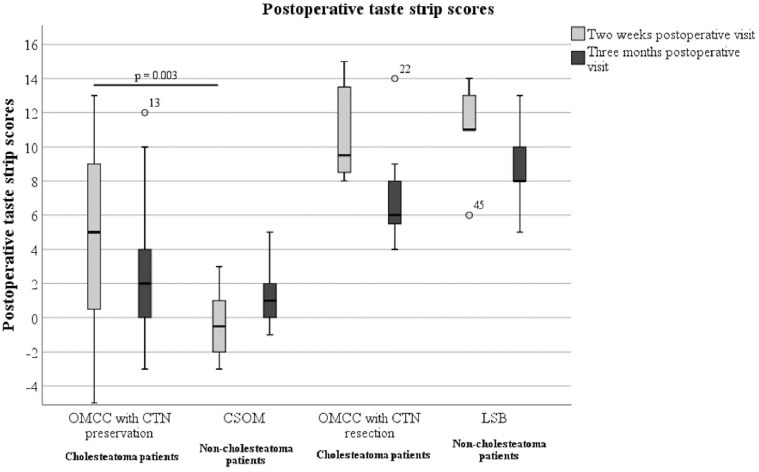
The 2 weeks postoperative and 3 months postoperative taste strip scores of all groups. Abbreviations: CSOM, chronic suppurative otitis media; CTN, chorda tympani nerve; LSB, lateral skull base lesions; OMCC, chronic otitis media with cholesteatoma.

The taste strip scores of OMCC patients with CTN resection were compared to those of LSB patients with CTN resection. Two weeks postoperatively, LSB patients (mdn = 11.0) showed higher taste strip scores than OMCC patients with CTN resection (mdn = 9.5). This difference did not prove to be significant (*P* = .833). Three months after surgery, the taste strip scores of OMCC patients with CTN resection (mdn = 6.0) and of LSB patients (mdn = 8.0) had decreased. No difference was seen between the groups (*P* = .284). The postoperative taste strip scores are shown in Figure 3.

*Subjective taste perception*: The postoperative results of the questionnaire are shown in Table 2b. Two weeks after surgery, a few OMCC patients with CTN preservation reported taste alteration (4/19 [21.1%]). Three months postoperatively, taste alteration persevered among OMCC patients with CTN preservation (4/17 [23.5%]).

**Table 2b.** Postoperative Results of the Taste Questionnaire.

	Time point	1-Year and long-term taste alteration (n [%])	Phanto-/parageusia (n [%])	Combination of these taste changes (n [%])
Cholesteatoma patients				
OMCC with CTN preservation	2 weeks (n = 19)	4 [21.1%]	2 [10.5%]	5 [26.3%]
	3 months (n = 17)	4 [23.5%]	3 [17.6%]	3 [17.6%]
OMCC with CTN resection	2 weeks (n = 8)	1 [12.5%]	1 [12.5%]	1 [12.5%]
	3 months (n = 8)	1 [12.5%]	1 [12.5%]	1 [12.5%]
Non-cholesteatoma patients				
CSOM	2 weeks (n = 14)	2 [14.3%]	1 [7.1%]	3 [21.4%]
	3 months (n = 13)	3 [23.1%]	—	2 [15.4%]
LSB	2 weeks (n = 5)	3 [60%]	—	1 [20%]
	3 months (n = 5)	1 [20%]	—	2 [40%]

The results of the taste questionnaire after surgery are shown as numbers and percentages.

Abbreviations: CSOM, chronic suppurative otitis media; CTN, chorda tympani nerve; LSB, lateral skull base lesions; n, number; OMCC, chronic otitis media with cholesteatoma.

Two weeks postoperatively, more than half of LSB patients noticed taste alteration. In contrast, only about 13% of OMCC patients with CTN resection experienced taste alteration. Three months after surgery, taste alteration persisted in 1 OMCC patient with CTN resection (1/8 [12.5%]). Less LSB patients described taste alteration (1/5 [20%]).

### Measured Taste Function Over Time

**Figure 4.**
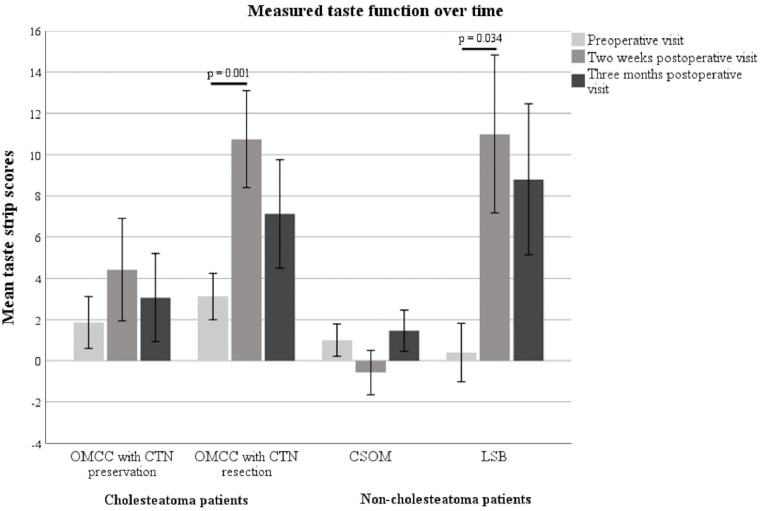
The mean taste strip scores of each group at the 3 measurement points. Abbreviations: CSOM, chronic suppurative otitis media; CTN, chorda tympani nerve; LSB, lateral skull base lesions; OMCC, chronic otitis media with cholesteatoma.

The 3 measurements of taste strip scores before and after surgery were compared for each group (Table 3, Figure 4). OMCC patients with CTN preservation showed no significant change of taste strip scores before and after surgery (*P* = .153). Neither did the CSOM patients (*P* = .057). A difference was observed between the preoperative and the 2 weeks postoperative taste strip scores of OMCC patients with CTN resection (*P* = .001). However, neither the preoperative compared to the 3 months postoperative taste strip scores (*P* = .182) nor the 2 weeks compared to the 3 months postoperative taste strip scores (*P* = .182) showed a significant difference. Similarly, LSB patients displayed a difference between the preoperative and the 2 weeks postoperative taste strip scores (*P* = .034). No difference was seen between the preoperative compared to the 3 months postoperative taste strip scores (*P* = .081) or between the 2 weeks compared to the 3 months postoperative taste strip scores (*P* > .999).

**Table 3.** Measured Taste Function Over Time.

Time point	Cholesteatoma patients		Non-cholesteatoma patients	
	OMCC with CTN preservation	OMCC with CTN resection	CSOM	LSB
Pre op	mdn = 2.0	mdn = 3.5*	mdn = 1.0	mdn = 0.0*
Two week	mdn = 5.0	mdn = 9.5*	mdn = −0.5	mdn = 11.0*
Three month	mdn = 2.0	mdn = 6.0	mdn = 1.0	mdn = 8.0

The median taste strip scores before and after surgery are presented.

Abbreviations: CSOM, chronic suppurative otitis media; CTN, chorda tympani nerve; LSB, lateral skull base lesions; mdn, median; OMCC, chronic otitis media with cholesteatoma; pre op, preoperatively.

* *P* < .05.

### Extension of the Cholesteatoma

All OMCC patients showed epitympanic extension of the cholesteatoma. Eleven (52.4%) of 21 OMCC patients with CTN preservation showed limited extension of the cholesteatoma and 10 (47.6%) patients showed moderate extension. The extension of the cholesteatoma and the preoperative taste strip scores did not correlate (*P* = .864). One (12.5%) of 8 OMCC patients with CTN resection showed limited extension, 5 (62.5%) showed moderate extension, and 2 (25%) showed extensive disease. The extension of the disease did not correlate to the preoperative taste strip scores (*P* = .655).

### Histological Examination

**Figure 5.**
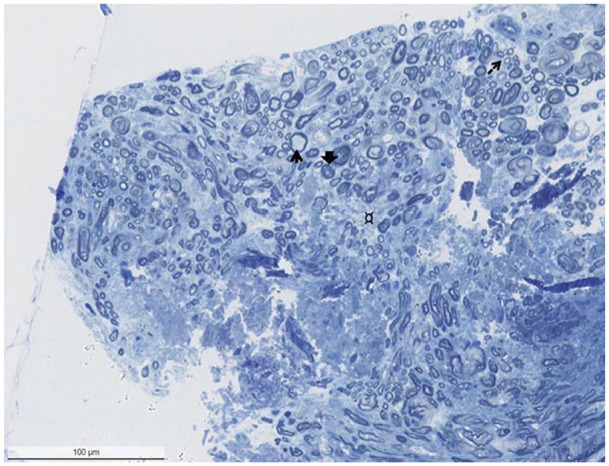
Biopsy showing mixed axonal and demyelinating neuropathy with extensive loss of large myelinated axons, accompanied by increased endoneurial connective tissue (¤), thinly myelinated fibers (thin arrow), and axonal atrophy (larger arrow). In addition, regeneration clusters can be seen (arrow with broken line). There is no evidence of inflammation (1 μm, toluidine blue-stained section).

Thirteen CTNs were resected. Three CTN specimens of OMCC patients and all 5 specimens of LSB patients could not be evaluated due to incorrect preservation, too little sample material, or extensive, superimposing artifacts. Two specimens of OMCC patients showed chronic axonal neuropathy characterized by axonal atrophy, degeneration, and fibrosis (Figure 5). One sample showed chronic mixed axonal neuropathy and demyelination. Two specimens displayed only mild axonal loss.

## Discussion

This study demonstrates an impaired measured taste function of OMCC patients before surgery. Subjectively this was often unnoticed. After surgery, both subjective taste perception and measured taste function decreased in OMCC patients. However, even after severing of the CTN, OMCC patients rarely reported taste alteration.

### Preoperative Considerations

Several studies of CTN specimens from patients with chronic otitis media have reported ultrastructural changes.^13,14^ These changes might result in loss of nerve function and, therefore, reduction of measured gustatory function. The current analysis of CTN specimens found chronic axonal neuropathy, which might explain the reduction of measured taste function in cholesteatoma patients compared to non-cholesteatoma patients before surgery. Measured taste function seemed to be influenced by the cholesteatoma; yet subjectively taste was perceived differently. Only a few cholesteatoma patients reported taste alteration preoperatively. A possible reason for this might be the chronicity of the disease. Since it is considered a gradually expanding, destructive epithelial lesion of the temporal bone, its effects on the CTN might not result in an immediate decrease of taste perception, with patients possibly adapting to the changes.^15^ Furthermore, typical cholesteatoma symptoms such as hearing loss, otorrhea, or otalgia might be more limiting for patients and therefore more noticeable.^16^

The cholesteatoma was more extensive in patients with CTN resection. This might lead to the evident conclusion that the more extensive the disease, the more likely the nerve has to be sacrificed. Yet the extension of the cholesteatoma did not correspond to the preoperatively measured taste function. Sano et al^17^ postulated that the damage to the CTN depended on the extent of direct invasion rather than on type of chronic otitis media. In the current study, the extension of the cholesteatoma was rated by classification of surgically challenging locations. Therefore, the extension of the disease might not adequately describe the extent of CTN destruction.

### Postoperative Considerations

Regarding measured taste function, middle ear surgery had a greater effect on OMCC patients with CTN preservation than on CSOM patients. This finding could be attributed to the different surgical approaches. In cholesteatoma surgery, the primary goal of surgery is complete removal.^18^ When necessary, the nerve is cleared from disease and thereby at risk for iatrogenic trauma (stretching, desiccation or injury). This might lead to more postoperative traumatization of the nerve compared to patients operated on for CSOM, where the CTN is hardly touched. Three months after surgery, the difference in measured taste function could no longer be detected, suggesting a recovery of nerve function in OMCC patients with CTN preservation. In both groups, no significant change of measured taste function over time was seen, and at the last visit, it was equal to the baseline. This might also suggest that nerve function recovers. Similarly, Saito et al^3^ observed a recovery of taste function in a majority of patients. In a study of CTN preservation during non-inflammatory ear surgery; it was observed that patients with a stretched CTN were more symptomatic than patients with a sacrificed nerve.^19^

CTN removal seemed to influence measured taste function of LSB patients more than of OMCC patients with CTN resection. However, this difference was not significant. Considering this, as nerve function of OMCC patients with CTN resection might have been impaired preoperatively, taste processing might have already adapted in these patients. As none of the lateral skull base lesions extended to the middle ear, the sudden loss of afferent fibers for gustatory innervation might have had a greater affect on LSB patients. In spite of considerable reduction of measured taste function after CTN removal, few OMCC patients with CTN resection reported taste alteration. Furthermore, 3 months after surgery taste perception of LSB patients seemed to improve. An explanation might be that taste is mediated via multiple nerves. The CTN must normally inhibit the glossopharyngeal nerve.^20^ When the CTN is blocked or damaged this inhibition is lost. This leads to perception of increased taste intensities from areas innervated by the glossopharyngeal nerve, thereby preserving a normal taste perception.^21^ As gustatory perception does not seem to be affected to a greater extent, this might suggest that in case of uncertainty, it is more favorable to sever the CTN in order to reduce the risk of cholesteatoma recurrence.

### Limitations

A limitation of this study is the possibility of damage to the CTN or the intermediate nerve due to a lateral skull base pathology. This could lead to distortion of preoperative taste function. However, preoperatively none of the LSB patients showed a reduced measured gustatory function or decreased facial function and the disease did not extend to the middle ear. Another limitation is the small number of patients in the groups with CTN resection. This might cause overestimation of the effect of these findings.

## Conclusions

Before surgery, cholesteatoma patients showed an impaired measured taste function compared to patients without cholesteatoma (CSOM, LSB). Subjectively this was often unnoticed. After surgery, despite removal of the CTN and consequent reduction of measured taste function, few patients reported taste alteration and subjective taste perception was even seen to be improving. In regards to middle ear surgery, perceived taste function does not seem to reflect measured gustatory function.
